# Pyoderma gangrenosum: pathogenetic mechanisms and their implications for treatment

**DOI:** 10.1007/s00281-025-01064-7

**Published:** 2025-10-23

**Authors:** Chiara Moltrasio, Maurizio Romagnuolo, Gianluca Tavoletti, Carlo Alberto Maronese, Angelo Valerio Marzano

**Affiliations:** 1 Dermatology Unit, Fondazione IRCCS Ca’ Granda Ospedale Maggiore Policlinico, Via Pace, 9, Milan, 20122 Italy; 2 Department of Medical Biotechnologies, University of Siena, Siena, 53100 Italy; 3 Department of Pathophysiology and Transplantation, Università degli Studi Di Milano, Milan, Italy

**Keywords:** Pyoderma gangrenosum, Pathogenesis, Pathogenesis-driven treatments, Medical management, Therapeutic algorithm

## Abstract

Pyoderma gangrenosum (PG) is a rare inflammatory skin disease belonging to the group of neutrophilic dermatoses. The pathogenesis of PG involves a predisposing genetic background that facilitates a dysregulated innate and adaptive immune response, with an imbalance between pro-inflammatory and anti-inflammatory mediators, leading to neutrophil-driven inflammatory damage. Several immunosuppressants and immunomodulatory drugs are currently available for the treatment of PG, in combination with topical therapies, wound management and pain control strategies. Systemic corticosteroids and cyclosporine remain the first-line treatment options with the best evidence. However, in recent years, the rise of knowledge about different pathogenic mechanisms has led to a significant increase in studies attesting the efficacy and safety of biologic therapies including, among others, antagonists of tumour necrosis factor (TNF)-α and interleukin (IL)-23, becoming the drug of choice in specific clinical setting. Similarly, different small molecules such as JAK-STAT (Janus kinase/signal transducer and activator of transcription) inhibitors are showing promising results for the treatment of PG. We review established and emerging pathogenesis-driven treatments, also providing a therapeutic algorithm and informing future directions in the management of PG.

## Clinical features

### Pyoderma gangrenosum clinical variants

PG initial description is attributed to Louis Brocq who, in 1908, observed ulcerative lesions characterized by a geometrical and irregular ridge, with an erythematous and infiltrated external slope and undermined internal slope of the lesion border. Brocq termed the disease “phagédenisme géométrique”, highlighting with this definition both the geometrical pattern and the rapidly necrotizing feature of these ulcers [1].

The current term “pyoderma gangrenosum” was coined by Brunsting and colleagues which described PG classical form as irregular ulcerations with well-demarcated erythematous-violaceus serpiginous borders and atrophic scarring, occasionally associated to infections or inflammatory bowel diseases [2].

Since these classical descriptions, additional clinical characteristics have been described and the current clinical classification of PG include different variants.

i) *Classic ulcerative* PG is the most common variant, initially presenting as a tender erythematous inflammatory papule, pustule or vesicle, which eventually starts to expand peripherally and degenerate centrally to produce a painful ulcer. PG ulcer border usually appears violaceous and undermined, while its base is usually necrotic and purulent. In terms of depth, ulcers in classic PG may erode deeply: they generally extend into the subcutaneous tissue and sometimes even further, exposing muscles and tendons. Patients may present with an isolated lesion or with multiple lesions in different phases of development, sometimes coalescing to form larger ulcers. The lower limbs tend to be the most frequently affected site, but lesions may occur in any body area. Finally, lesions in classic PG usually heal by second intention with a characteristic atrophic cribriform scarring (termed Gulliver sign) and a “cigarette paper-like” appearance [3].
ii) *Pustular* PG is characterized by the acute onset of discrete pustular lesions with an erythematous halo, mainly involving the trunk and the extensor surfaces. These pustules may then either resolve or progress to form ulcers. Associated fever and arthralgias are frequent. This form is most common amongst patients also suffering from inflammatory bowel disease (IBD) and usually appears during acute exacerbations of the condition [4].
iii) *Bullous* PG, also known as atypical PG, represents a superficial form of pyoderma gangrenosum. Patients usually develop painful haemorrhagic vesicles or bullae with blue-gray inflammatory borders, rapidly progressing to superficial ulcers. The most frequently involved site of such lesions are the upper extremities (including dorsal hand) and the face. A significant association between bullous PG and hematological malignancies is recognized – in up to 70% of the reported cases – thus requiring a thorough evaluation for the concomitant presence of hematological disorders [3, 5].
iv) *Vegetative* PG (also known as superficial granulomatous type) unlike other variants, has a gradual onset, slower progression, and only causes mild discomfort. It presents as a non-purulent erosion or superficial ulcer without the classical erythematous to violaceous undermined borders. They most frequently appear on the trunk and often exhibit a verrucous surface [3, 6].

### PG in uncommon locations

Apart from the four above-mentioned clinical subtypes, two other atypical variants of PG related to surgical procedures and associated to local inflammatory stimuli and pathergy have been recognized:

(i) *Post-operative* PG, also known as pathergic PG or progressive gangrene of Cullen named from the first surgeon who described it in 1924 represents a rare complication of a wide range of surgical intervention and is characterized by the acute onset of PG-like lesions within surgical sites [7]. The most common location seems to be the mammary area with a classical sparing of the nipple-areolar complex, although it has been described following a variety of surgeries including cardiothoracic, gynaecological and orthopaedic interventions [8]. The lesions typically appear after 7 to 11 days after the surgery with painful erythema that rapidly necrotize with small ulcers that could coalesce to form larger ulcerations; wound dehiscence is common. In these cases, differentiating post-operative PG from necrotizing fasciitis is crucial, as wound debridement could worsen a PG-like eruption due to pathergy phenomenon [7]. Interestingly the majority of the patients (66% in a recent review) do not present an associated comorbidity as compared to classical PG (50% to 75% of associated diseases), and the lesions usually respond to immunosuppressive therapies [8].
(ii) *Peristomal* PG occurs in the skin around sites of ileostomy or colostomy as a classical ulcerative PG [8]. In around 80% of the cases patients have an associated IBD and PG occurs in a variable time – ranging from 1.5 to 23 months - after stoma creation [8, 9]. Peristomal PG is more common in women and usually follows an IBD flare, however it could present also in patient with no evidence of IBD [9]. A prompt diagnosis is crucial for the correct management of this condition and to differentiate it from other stoma-related complications, in order to avoid unnecessary treatments as for the post-operative PG. Local and systemic immunosuppressive treatments are usually effective but, interestingly, stoma replacement and/or closure has been associated with resolution of peristomal PG [9].

In addition to PG clinical subtypes and localized post-surgical variants, special considerations should be given to paediatric forms and PG involving uncommon anatomical sites (e.g. ano-genital, head-and-neck area and mucosae).

*Pediatric* PG accounts for approximately 4% of the PG cases and differently from adult-onset PG tends to present with more than one ulceration and a slight predilection for the head-and-neck area, with a higher reported rate of pathergy (around 62% in children compared to 25–30% in adults); in infants and toddler ano-genital involvement was also reported. Pediatric PG equally affects male and female, being idiopathic in half of the reported cases; IBD still represent the most common association (around 20% of the cases) while haematological disorders are less frequently reported compared to adults (8% in children compared to 15–25% in adults) [10].

Head-and-neck (HN) involvement in PG is rare and usually presents with atypical clinical features: pustular or ulcerating nodules over the face represents a common presentation of this form compared to classical PG; pathergy rates (around 37% of the patients) and recurrence risk (approximately 15%) are higher in this form compared to classical PG. Of note 20% of the patients with HN PG had a previous diagnosis of PG or unspecified ulcers. Ulcerative colitis represents the most common associated diseases in HN PG as opposed to Crohn’s diseases in classical PG. Local tissue destruction and eye involvement is a well-described complication of HN PG, occurring in approximately a third of patients. These features could help clinicians in the differential diagnosis with other ulcerative diseases in the head-and-neck area including granulomatosis with polyangiitis, vasculitis and cancers, although the clinical diagnosis remains challenging [11].

Mucosal involvement of the oral cavity in PG is extremely rare and could affect the tongue, soft and hard palate, lips, buccal mucosa, gingiva and tonsillar fauces, presenting as painful and sometimes bleeding irregularly shaped ulcers with raised borders; initially a papule or nodule could be observed as in classical PG. Males appear more frequently (65%) affected than women and in 20% of the cases PG cutaneous involvement is not present. An associated systemic disease is detected in the majority of the patients (80%), with Crohn’s disease representing the most common comorbidity. Differential diagnosis of oral PG is broad, especially in the absence of PG cutaneous lesions, as it could mimic several oral ulcerative conditions including infective, inflammatory and neoplastic diseases [12].

Ano-genital involvement in PG is uncommon, and pose major challenges in differential diagnoses as for the head-and-neck area [13]; a retrospective study observed a median age of onset of 43 years, with the most affected areas being vulva, penis and perineum; usually, one the three ulcerations were present, exclusively located in the ano-genital area in the majority of the studied patients. Classical ulcerative and post-operative PG were the widely reported clinical variants. IBD and haematological malignancies were associated to ano-genital PG, however in lower rates compared to PG in other anatomical locations [13]. An interesting review study identified a strong association between vulvo-vaginal PG and rituximab exposure: all the female reported in the study (14 patients) developed PG in the vulvo-vaginal area after rituximab administration at standard doses for different diseases (neoplastic or autoimmune); moreover, rituximab discontinuation led to PG resolution in the majority of the cases (75%) [14].

Indeed, a minority of PG cases may be drug-induced. Adopting the Naranjo criteria for adverse drug reactions, cocaine/levamisole was identified as a probable cause of PG [15], although distinguishing true PG from PG-like lesions in levamisole-induced ANCA + vasculitis may be challenging. Other medications possibly linked to PG onset or exacerbations were isotretinoin, propylthiouracil, and the tyrosine-kinase inhibitors sunitinib and gefitinib [15]. PG may also occur as a paradoxical reaction following IL-17 inhibition [16], while its appearance after anti- tumour necrosis factor (TNF)-α agents, albeit reported, is still under debate [15, 17].

#### Syndromic forms

In a more restricted subset of patients, PG represents a component of distinct autoinflammatory syndromes [18], whose prototype is PAPA (pyogenic arthritis, PG and acne), hallmarked by sterile cutaneous inflammation and arthritis and caused by pathogenic variants in *PSTPIP1* (proline-serine-threonine phosphatase interacting protein 1) gene that lead to an altered proinflammatory signaling and neutrophil-driven inflammation [18].

In recent years, the spectrum of autoinflammatory diseases caused by *PSTPIP1* mutations or characterized by clinical features similar to those seen in PAPA syndrome has been expanding, encompassing several entities [18, 19] as summarized in Table 1.

**Table 1:** Pyoderma gangrenosum-associated autoinflammatory syndromes

Pyoderma gangrenosum, acne, pyogenic arthritis (PAPA)
Pyoderma gangrenosum, acne, suppurative hidradenitis (**PASH**)
Pyogenic arthritis, pyoderma gangrenosum, acne, suppurative hidradenitis (**PAPASH**)
Psoriatic arthritis pyoderma gangrenosum, acne, suppurative hidradenitis (**PsAPASH**)
Pustular psoriasis, arthritis, pyoderma gangrenosum, synovitis, acne and suppurative hidradenitis (**PsAPSASH**)
Pyoderma gangrenosum, acne, suppurative hidradenitis, ankylosing spondylitis (**PASS**)
Pyoderma gangrenosum, acne, ulcerative colitis (**PAC**)
Psoriatic arthritis, pyoderma gangrenosum, suppurative hidradenitis, crohn’s disease (**PsAPSC**)
Vasculitis, pyoderma gangrenosum, acne, suppurative hidradenitis (**VPASH**)
PSTPIP1-associated myeloid-related-proteinemia inflammatory syndrome (**PAMI**)

### Diagnostic tools and work-up

Although many efforts spent in proposing specific and validated diagnostic criteria, PG remains a diagnosis of exclusion and the integration of clinical, anamnestic and laboratory data is crucial for the final diagnosis. Indeed, no laboratory or histopathological marker exists for this condition.

Table 2 summarizes the three main proposed diagnostic criteria for PG [5, 20, 21]. These three diagnostic criteria (Su and colleagues [5], Delphi consensus [20] and PARACELSUS [21]) have been compared for diagnostic accuracy of PG; PARACELSUS accurately scored 89% of PG patients, followed by Delphi Consensus and Su and colleagues, which each assessed 74% of patients with PG [22].

**Table 2:** Overview of proposed diagnostic scoring systems for pyoderma gangrenosum

Authors, year, reference	Scoring and diagnostic criteria
Su et al., 2004 [5]	Both major + ≥ 2 minor criteria = PG • Major: ulcer morphologically consistent with PG; exclusion of other causes. • Minor: pathergy or cribriform scarring; systemic disease associated with PG; histopathologic findings compatible to PG; response to immunosuppression.
Delphi Consensus, 2017 [20]	Major criterion + ≥ 4 minor criteria = PG. • Major: biopsy of ulcer edge demonstrating neutrophilic infiltrate • Minor: - exclusion of infection; - pathergy; - history of inflammatory bowel disease or inflammatory arthritis; - history of papule, pustule, or vesicle ulcerating within 4 days of appearing; - peripheral erythema, undermining border, and tenderness at ulceration site; - multiple ulcerations, at least 1 on an anterior lower leg; cribriform or “wrinkled paper” scar(s) at healed ulcer sites; - decreased ulcer size within 1 month of initiating immunosuppressive medication(s).
PARACELSUS Score, 2019 [21]	≥ 10 points = PG likely. Major (4 points): rapid progression; exclusion of other causes. • Minor (2 points): pathergy; associated systemic disease; consistent histopathology; rapid response to therapy. • Additional (1 point): pain; multiple ulcers; cribriform/vegetative scarring; undermined border.

PG differential diagnosis is broad and could be divided in six main categories: (i) infection (bacterial, viral, fungal, protozoal); (ii) systemic vasculitis and vasculopathy, (iii) neutrophilic dermatosis (e.g. Sweet syndrome, subcorneal pustular dermatosis and bullous lupus erythematosus); (iv) vascular; (v) neoplastic and (vi) exogenous tissue injury as reviewed by Weenig and colleagues [23].

In Table 3, diagnostic work-up for PG diagnosis wassummarized.

**Table 3:** Diagnostic work-up for pyoderma gangrenosum

Step	Assessment	Clinical Relevance
1. Anamnesis and physical examination	Identify rapidly progressive, painful ulcers with undermined violaceous borders. Full body examination is warranted. Inquire into history of recent traumas or surgeries (pathergy phenomenon). Screen for associated systemic diseases (IBD, arthritis, hematologic disorders) and drug history	Clinical suspicion is essential. Up to 75% of PG cases have comorbidities. Pathergy positive in up to 30%.
2. Histopathology	Elliptical incisional biopsy or a deep punch biopsy, which includes the lesion’s inflamed border and ulcer edge, and penetrates vertically into the subcutaneous tissue.	Histology is not pathognomonic but is essential in differential diagnosis. Rule out an infectious disease
3. Laboratory work-up	CBC with differential, ESR, CRP, liver and renal function tests, autoimmune panel (ANA, ANCA, RF), serum protein electrophoresis, immunoglobulins. Hypercoagulability studies (e.g. antiphospholipid antibodies). Microbiological swabs/cultures and infectious disease screening. Additional tests according to suspected comorbidity (e.g., calprotectin for IBD).	Laboratory findings are non-specific but support differential diagnosis and screen for associated diseases.
4. Exclusion of alternative diagnoses	Rule out infection, vasculitis, neoplasia, vascular, and drug-induced ulcers. Correlate clinical, laboratory, microbiological, and histological findings.	PG remains a diagnosis of exclusion.
5. Additional assessments (if indicated)	Imaging (Doppler US, Chest radiography, MRI/CT for deep involvement/osteomyelitis or neoplasia). EGDS, colonoscopy.	Age and symptoms appropriate oncological screening should be considered.
6. Ongoing re-evalutation	Reassess diagnosis if lack of response. Monitor for new comorbidities. Consider alternative diagnosis if non-responsive.	Diagnosis may evolve over time, especially if initial treatment fails.

*PG* (Pyoderma Gangrenosum), *IBD* (Inflammatory Bowel Disease), *CBC* (Complete Blood Count), *ESR* (Erythrocyte Sedimentation Rate), *CRP *(C-Reactive Protein), *ANA* (Antinuclear Antibody), *ANCA* (Antineutrophil Cytoplasmic Antibody), *RF* (Rheumatoid Factor), *US* (Ultrasound), *MRI *(Magnetic Resonance Imaging), *CT* (Computed Tomography),* EGDS* (esophagogastroduodenoscopy)

### Histopathology of PG

As mentioned above, PG histopathology is not specific and requires clinical correlation. The major findings in active lesions of the classical ulcerative variant are represented by a tissueneutrophilia with epithelial undermining and ulceration, occasional dermal edema and vascular damage with fibrinoid necrosis, and no evidence of leukocytoclastic vasculitis or bacterial, mycobacterial and fungal infections. Neutrophils are often folliculocentric, and a central unspecific area of suppurative necrosis is often present; an accompanying perivascular and intramural lymphocytic infiltrate with scattered neutrophils is also characteristic [3, 24]. With regards to the other clinical variants of PG, bullous subtype presents with an extensive epidermal necrosis with neutrophilic subcorneal, epidermal and/or subepidermal bullae formation joint with a dense dermal neutrophilic infiltrate [3, 24]. Vegetative PG presents with pseudoepitheliomatous epidermal hyperplasia and a dermal palisading granulomatous reaction with neutrophilic abscesses and sinus tracts [3, 24]. Pustular PG variantsare characterized by a subcorneal neutrophil accumulation and/or a follicular and perifollicular neutrophil accumulation with suppurative features [3, 24]. PG cases associated with Crohn’s disease may exhibit granulomatous features [24].

The appropriate clinical setting and clinical correlation could differentiate PG lesions from its histopathological mimicker, i.e., Sweet syndrome (which is rarely folliculocentric), a severe vesicular-necrotic insect bite reaction, a pustular drug reaction, and neutrophilic folliculitis in the setting of rheumatic diseases [24]. As stated above, an appropriate biopsy should be performed on the active ulcer edge and reach the deep dermis and subcutaneous tissue; the timing of biopsy is also fundamental, as very incipient lesions or older/resolving ulcers may show non-specific histopathological findings [24].

Making the diagnosis in such instances represents a challenge of utmost therapeutic importance, as reliance on biopsy results should limit or delay treatment initiation. Rather, a practical, ex-adjuvantibus approach may be encouraged, especially if infectious causes have been excluded.

### Scoring systems and patient reported outcomes

Given the rarity of PG, there are no currently validated or widely available outcome measures, both clinical and patient reported, for this disease. This lack in validated measurement could affect patient’s management in clinical practice and clinical trials interpretability leading to limitations in newly therapeutic development and evidence-based treatment [25].

Though a specific quality of life (QoL) questionnaire for PG is not available, the psychological and social burden of the patient is high, as demonstrated by the high scores in Dermatology Life Quality Index (DLQI) and the frequent association with depression [3]. The impact on quality of life is multifaceted, and an interview analysis identified 8 domains which were critically affected and differentiated by PG patients: pain, physical limitations, hygiene, self-image, mental health, sexual intimacy, wound care and disease course [26]. Among all the domains pain seems to be the most important factor in deteriorating QoL, and improvements in pain numerical rating scale (NRS) could correlate with the physician global assessment (PGA) score [3, 27].

Recently an effort to provide a core set domain for PG to be used in interventional clinical trials was promoted on behalf of the UPGRADE initiative, which through a rigorous process involving experts, patients, researcher and industries identified three main domains to be included in the core set: (i) pain, (ii) quality of life and (iii) clinical sign [25].

Validated PG-specific instruments to measure these domains will be the next step of the consensuswork [28].

In the meanwhile, the 5-point Global Pyoderma Gangrenosum (GPG) Severity Score has been proposed for the upcoming phase III trial on the Interleukin (IL)−36 receptor inhibitor spesolimab (NCT06092216) [29].

## Epidemiology

The exact epidemiology of PG remains difficult to estimate since only a few population-based studies have been conducted so far. In addition, the difficulty in correctly diagnosing PG might impair the accuracy of estimates.

One of the first studies conducted in UK showed an incidence rate of about 6 per million person-years with a median age at PG presentation of 59 years and no gender predominance [30]. The largest cross-sectional study from the USA recently reported a worldwide incidence of PG estimated at 3–10 cases per million people with a prevalence of about 58 per million adults-years. Patients over 50 years of age accounted for nearly 70% of all PG cases with a female-to-male ratio of >1.8 [31, 32]. Associated diseases have been found in approximately 50 to 75% of PG patients. Epidemiological data are similar across US and different European countries [3].

It has also been reported that PG patients have a significantly increased mortality rate compared with those of the general population, including a study that found a three folds higher mortality for PG patients compared to age and sex matched controls [3, 30].

## Etiology

The etiology of PG is multifactorial, encompassing physical trauma, pharmacologic triggers, and coexisting systemic diseases. These factors are thought to interact with dysregulated immune responses, potentially initiating or exacerbating lesion formation; however, the exact pathogenic mechanisms remain incompletely understood [30].

As mentioned above, a phenomenon commonly associated with PG is the pathergy reaction—a hyperreactive response to minor skin trauma, resulting in new lesions formation at sites of injury. Pathergy manifests in about 30% of PG cases [7] with even minimal trauma, such as needle punctures, insect bites, or surgical incisions, capable of inducing ulcers in predisposed individuals [33].

In addition to the previously mentioned role of different pharmacological agents acting as potential triggers [15], PG may also manifest during pregnancy or the postpartum period, phases characterized by elevated neutrophil counts and systemic inflammation, which could exacerbate PG in susceptible individuals [34].

A significant etiological component of PG is its frequent association with systemic diseases, reported in up to 50% of cases. The most common associations are with IBD, arthritis, and hematologic disorders [35]. Clinical observations often indicate that these systemic diseases precede the onset of PG. Specifically, IBD is present in 15–30% of patients with PG, whereas PG develops in approximately 0.5–2% of individuals diagnosed with IBD [36]. PG activity may coincide with IBD flares but can also arise independently. Notably, the improvement of one condition occasionally corresponds with improvement in the other.

Arthritis is observed in approximately 10–20% of PG cases, most frequently presenting as rheumatoid arthritis or seronegative spondyloarthropathies, including ankylosing spondylitis and psoriatic arthritis. PG has additionally been associated with peripheral arthritis and various inflammatory joint diseases of unclear etiology [37].

Hematologic disorders associated with PG, present in 7–15% of cases, include monoclonal gammopathies—primarily IgA gammopathy—and hematologic malignancies such as acute myeloid leukemia and myelodysplastic syndromes. In PG patients, monoclonal gammopathies may progress to multiple myeloma; however, current evidence does not indicate a significantly higher progression rate than in cases without PG [38].

Importantly, monoclonal gammopathy of undetermined significance (MGUS) associated with PG should be thought of as a monoclonal gammopathy of cutaneous significance and, following careful discussion of the possible risks with the patient, may be treated actively.

PG has also been linked to other neutrophilic dermatoses and autoimmune conditions, such as subcorneal pustular dermatosis, Behçet’s disease, Sweet’s syndrome, and systemic lupus erythematosus (SLE) [39]. In addition to the well-established associations of PG with specific systemic diseases, multiple reviews have identified potential links with other conditions. The clinical relevance of these associations varies, and some are considered anecdotal due to limited evidence of a direct correlation. Among these, diabetes mellitus can complicate management of PG by impairing wound healing, increasing infection risk, and potentially exacerbating inflammation. The prevalence of diabetes among PG patients varies, with some studies indicating that up to 15% of patients may have concomitant diabetes [40].

## Pathogenesis

Available evidence on the pathophysiology of PG points towards a close interaction between neutrophil dysfunction, overexpressed inflammatory mediators and dysregulated components of both innate and adaptive immune response upon a predisposing genetic background, in which the follicular unit has been proposed as the initial target of subclinical inflammation [41].

Consistent with its classification within the spectrum of neutrophilic dermatoses (NDs), a wide range of data confirmed the role of neutrophils in PG pathogenesis. Indeed, tissue array analyses identified different overexpressed pro-neutrophils markers, including IL-8, chemokine (C-X-C motif) ligand (CXCL) 1/2/3/16 and RANTES (regulated on activation, normal T-cell-expressed and secreted) in PG lesional skin [42]. In PG, IL-8, also known as CXCL8, is secreted by several cell types such as dermal fibroblasts, endothelial cells, T lymphocytes and macrophages and acts as one of the major mediators of the inflammatory response functioning as a potent chemoattractant for neutrophils and angiogenic factor [42]. Similarly, TNF-α is a central pro-inflammatory cytokine and has been found to be overexpressed in both PG lesional tissue and serum [42]. Extensive crosstalk between these two inflammatory networks might favour a persistent neutrophil function and recruitment in PG tissue; furthermore, the release of IL-8 and TNF-α seems to be potentiated by CD40/CD40L and Fas/FasL system, both belonging to the TNF/TNF receptor superfamily and found to be increased in lesional PG [42]. Some evidence also reported a role of G-CSF (granulocyte colony-stimulating factor), a pleiotropic cytokine with specific effects on the proliferation, differentiation and activation of hematopoietic cells of the neutrophilic granulocyte lineage, in PG pathogenesis [43], although further mechanistic studies should be required.

Neutrophil activity is tightly regulated by three main strategies, (i) phagocytosis, (ii) degranulation and (iii) release of neutrophil extracellular traps (NETs). This latter are structures composed of DNA-histone complexes and anti-microbial proteins able to entrap bacteria, fungi, protozoa and virus as well as stimulate the release of proinflammatory mediators and cause vascular endothelium damage [44]. An enhanced formation of NETs has been reported in several immune-mediated inflammatory diseases, including PG and its syndromic form PAPA [45]. Interestingly, some evidence has also emphasized the presence of NETs with an enrichment of low-density granulocytes (LDGs) signature in PAPA lesional skin [45]. LDGs are a proinflammatory subset of neutrophils that express cell-surface markers of both terminally differentiated and immature neutrophils and are considered highly proinflammatory because of the increased spontaneous NETs formation and cytokine release [45]. Although there is no evidence yet, it is plausible that NETs/LDGs may contribute to the pathogenesis of sporadic PG, as previously demonstrated for other immune-mediated inflammatory conditions. Further confirmation of the pathogenic role of neutrophils comes from some recent molecular studies [46–48], in which signaling pathways linked to neutrophil-related inflammation and neutrophil homeostasis/activation/degranulation appeared to be nodal pathogenic drivers in PG pathogenesis. One of these studies [46] also revealed, in terms of differentially expressed genes (DEGs), an involvement of complement cascade within the perilesional dermis of PG patients. The complement-activated product, C5a, is a strong chemoattractant for different immune cells, including neutrophils, which in turn promote its function through a self-amplification process. Growing data suggest that C5a is a crucial bridge between innate and adaptive immunity, thus extending its role in inflammation; in PG, its blockage might ameliorate neutrophilic inflammation and promote ulcer healing.

In the pathogenic scenario of PG, one of the more interesting aspects lies in the recent evidence supporting that follicular adnexal structures may be the initial target [41, 49]. Following inflammatory priming in genetically predisposed individuals, perivascular and peripilosebaceous T-cells skew towards a T helper (Th)1/Th17 phenotype. Alongside Th17 activation and multiple interactions between the follicular/interfollicular epithelium and immune cells, a complex neutrophil-dominant autoinflammatory mileu, with elevated levels of TNF-α, IL-1 α/β, IL-8, IL-12, IL-15, IL-17, IL-22, IL-23 and IL-36 occurs. It has also been proposed that once that Th17 response is well-established, local Th9 cells mediate the release of IL-9, which in turn is downregulated by interferon (IFN)-γ through dendritic cells modulation of IL-27 [50].

Although the immune-inflammatory signature shows neutrophils as the principal actors during active ulcerative stage, a role of fibroblasts, monocytes/macrophages, natural killer (NK) and B-cells has also been assumed [51], although additional research is needed.

Th2 related molecules including IL-4, IL-5, IL-13 and C-C motif chemokine receptor (CCR) 3 have been found to be overexpressed in PG tissue compared with healthy controls (HCs). Since IL-4 and IL-5 are important mediators in B lymphocyte proliferation and differentiation, this may be indicative of B-cell involvement in PG pathogenesis [51]. At the same time, the marked reduction of Th2-mediators compared with the predominant Th1‐signature may underlie the aggressive course of the disease as well as refractoriness of PG lesions in the absence of immunosuppressive agents [52]. Furthermore, as previously described, the pathogenic role of NETs has been proved in different autoinflammatory diseases including, among others, hidradenitis suppurativa (HS). In the latter, Byrd et al., [53] in addition to an increased frequency of CD86 + B cells, plasma cells and IgG, found elevated autoantibodies reactive to different citrullinated proteins in NETs, thus hypothesizing that enhanced autoantigens generation, through increased NET formation, could play a role in promoting a dysregulated adaptive immune response. It has therefore been speculated that a similar mechanism may also occur in PG, although evidence is currently scarce [51].

The contributory role ofadaptive immunity was previously demonstrated by the presence of CD3 + T lymphocytes in the inflammatory infiltrate of PG’s wound edge [42] and T cell oligoclonality in response to some form of autoantigens [54]. Moreover, a reduction of regulatory T cells (Tregs) supported the hypothesis of a dysregulated T cell response, thus allowing an uncontrolled activation of effector T cells, such as Th17 cells [55].

It has been also demonstrated that activation of monocyte/macrophage cell lineage is able to produce IL-15, a key cytokine involved in wound repair and regeneration and found to be overexpressed in PG dermal perivascular regions [52]. IL-15 is a pleiotropic cytokine that plays an important role both in innate and adaptive immunity, protecting neutrophils from apoptosis, stimulating IL-8, TNF-a, IL-6 and IL-1 secretion and promoting lymphocyte and NK cell proliferation and function. As for IL-15, an increased expression of NK cell marker CD56 has been found in PG cell infiltration, suggesting the involvement of IL-15 in NK cell activation and proliferation [52]. Noteworthy, NK precursors (NKPs) acquire CD122 (IL2Rβ) expression, which is crucial in the signaling transduction of IL-15 via janus kinase (JAK)1/3 and signal transducer and activator of transcription (STAT)5. Therefore, the impairment or loss of one of these components precludes NK cell development, thus highlighting the important role of JAK/STAT pathway in NK cell maturation [56]. Differential expression of JAK signaling genes, mainly JAK3 and STAT4, has also been reported in a recent molecular study conducted by Ortega et al., finding further confirmation in previous evidence, particularly by that described by therapeutic effectiveness of JAK inhibitors in PG [46].

IL-15 mRNA is constitutively expressed not only in monocytes/macrophages but also in nonhematopoietic cells such as fibroblasts, whose role has been partially proven in PG pathogenesis [41]. Indeed, an interesting study demonstrated a significant decrease of CD4 + and CD34 + fibroblasts as well as IL-15 and desmin in PG wound healing compared with active ulcerative stage and unaffected skin. These findings, in addition to suggesting a potential pathogenic role of fibroblasts, revealed the destruction of pilosebaceous unit during PG resolution consistent with the almost complete absence of desmin [41].

Finally, it’s important to mention the role of genetic variants in this complex scenario. Indeed, in addition to the involvement of the “paradigmatic” gene *PSTPIP1* (serine-threonine phosphatase interacting protein 1), pathogenic variants in autoinflammatory genes such as *MEFV* (mediterranean familial fever), *NLRP* (NOD-like receptor family pyrin domain containing) 3/12, *NOD2* (nucleotide binding oligomerization domain containing 2 genes) and *IL1RN* (interleukin 1 receptor antagonist), leading to an exacerbated release of IL-1β, have been reported in both syndromic (PAPA, PASH and PAPASH) and sporadic PG [18]. More recently, other autoinflammatory genes such as *NCSTN* (nicastrin) and *NLRC4* (NLR family CARD domain containing 4) have been identified in PAPASH syndrome whereas causative variants in *OTULIN* and *GJB2* (gap junction protein beta 2), - the latter involved in keratinization pathway -, have been reported in PASH patients [57]. *AIM2* (absent in melanoma 2), a key pathogen sensor capable of initiating the assembly process of inflammasome and leading to the secretion of both bioactive IL-1β and IL-18, appears to play a role in susceptibility to syndromic forms of PG but not to sporadic PG [58], although validation studies are needed.

Noteworthy, a recent systematic review [59] with the objective of identifying inborn errors of immunity (IEis) coexisting with PG demonstrated an association between these conditions, reporting additional genetic variants potentially involved in PG pathogenesis. According to the authors, clarify the role of IEIs in the pathogenic context of PG may increase our understanding of the molecular pathomechanisms of this disease.

In conclusion, recent insights into the complex interaction between immune cells and inflammatory signature as well as the role of genetic predisposition has shed light on novel cellular and molecular mechanisms of PG, although further investigation is needed to fully unravel the pathogenesis of this complex disease and build promising therapeutic strategies in the era of personalized medicine.

## Pathogenesis-driven treatments

**Fig. 1:**
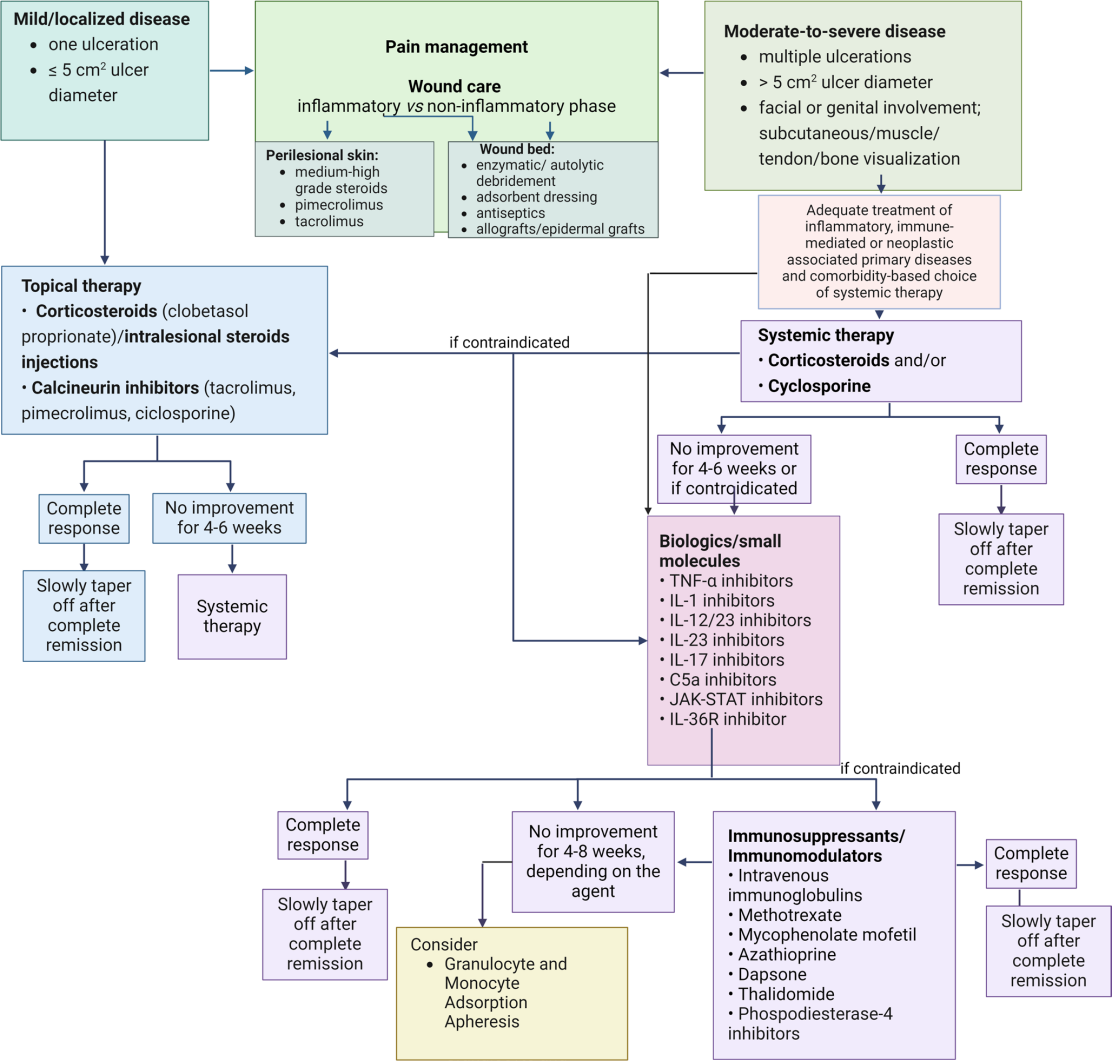
Therapeutic algorithm for the management of pyoderma gangrenosum. Created with BioRender.com TNF, Tumour Necrosis Factor; IL, interleukin; C5a, complement component C5a; JAK-STAT, Janus kinase/signal transducer and activator of transcription; R, receptor

Several immunosuppressants and immunomodulatory agents are currently available for affected patients, in combination with topical pharmacologic therapies, wound management and pain control strategies. However, in recent years, a significant increase of studies has proven the efficacy and safety of biologic therapies and small molecules blockers, which is why the authors are proposing an updated therapeutic algorithm (Fig. 1).

### Level 1 evidence (Clinical trials data)

#### Systemic corticosteroids

Corticosteroids (CS) represent an excellent first-line treatment choice for PG worldwide. They exert their immunosuppressive action by affecting transcription factors, thus acting on the regulation of immune-related genes. They promote a reduction of proinflammatory cytokines such as IL-1 and TNF-α, chemokines and cell-adhesion molecules while increasing anti-inflammatory mediators such as IL-10 and T-regs, thus leading to a decreased neutrophil chemotaxis and migration to inflammation sites [60].

The STOP-GAP (Study of Treatments fOr Pyoderma Gangrenosum Patients) randomised controlled trial showed that prednisolone at a dosage of 0.75 mg/kg/d resulted in clinical remission in up to half of PG cases by 6 months, although about a third of these patients developed recurrence after completing the study [61]. Patients with milder disease, in the absence of underlying comorbidities, achieved a better clinical response to CS and steroid-sparing agents should be started concomitantly to allow tapering once disease remission is achieved [61].

The use of CS should be discouraged in patients suffering from obesity, osteoporosis, gastrointestinal ulcers and/or mental illness. Serious adverse events (SAEs) related to CS including acute kidney injury, hyperglycemia, drug-induced diabetes, cellulitis, and bowel perforation have been reported in clinical practice [62].

#### Calcineurin inhibitors (cyclosporine and tacrolimus)

Cyclosporine and tacrolimus are calcineurin inhibitors that impairs the transcription of different cytokine genes such as IL-2 and IFN-γ, particularly in activated T cells.

In the STOP-GAP trial, a comparison of prednisolone (0.75 mg/kg/d) and cyclosporine at the dosage of 4 mg/kg/d found no difference in healing rates (47%) of PG lesions by 6 months, inflammation resolution and time to recurrence; in this regard, about 28% of patients treated with cyclosporine and 30% of patients treated with prednisolone had a recurrence after a median of 582 days [61].

Compared to CS, cyclosporine had fewer SAEs [62] and it was found to be a more-cost effective treatment option for PG, particularly for lesions of 20 cm2 or greater [63].

Oral tacrolimus was administered in several patients with satisfactory response, showing a greater potency than cyclosporine. Potential AEs were similar to that of cyclosporine but may be continued longer [62].

#### Tumor necrosis factor alpha inhibitors

The overexpression of TNF-α and its related cytokines in PG lesional skin has led to frequent administration of TNF-α antagonists including adalimumab, certolizumab pegol, etanercept, infliximab and golimumab for the treatment of PG. Complete remission was observed in 87% of PG patients with disease duration of less than 12 weeks, compared with 69% of those with disease duration of more than 12 weeks [62].However, higher response rates favour adalimumab and infliximab [64, 65]. Indeed, a phase III open-label study conducted by Yamasaki et al., [65] reported a response rate of 54.5% in subjects receiving adalimumab after 6 months of treatment; similarly, a randomized, double-blind, controlled trial conducted by Brooklyn et al., [64] demonstrated a clinical benefit in 46% of patients after 2 weeks of infliximab treatment compared to placebo and clinical improvement in 69% of PG patients by week 6.

Certolizumab pegol and golimumab have only anecdotal evidence in PG [62].

#### C5a inhibitors

The known role of C5a in neutrophil recruitment, wound healing, and complement dysfunction seen in PG lesional skin make it a promising therapeutic target for PG [46].

Vilobelimab is a first-in-class chimeric monoclonal antibody (mAb) that highly and effectively inhibits activity of complement factor C5a while leaving the formation of the membrane attack complex (C5b-9) intact as a defence mechanism of the innate immune system. A phase IIa open-label study testing the efficacy and safety of vilobelimab (IFX-1) (NCT03971643) [66] showed that 85.7% of PG patients achieved clinical remission with closure of the target ulcer as assessed by Physician’s General Assessment (PGA) score (equal to or less than 1.15). A phase III clinical study is currently underway to investigate its efficacy and safety in the treatment of PG [66].

Although no other trials with complement inhibitors are currently under investigation for PG, potential alternatives including (i) avacopan (formerly CCX168), an allosteric C5a receptor (C5aR) antagonist approved by Food and Drug Administration (FDA) for anti-neutrophil cytoplasmic (auto)antibody (ANCA)-associated vasculitis (AAV); (ii) eculizumab, a humanized mAb that was approved for UC and able to prevent the release of C5a and activation of the terminal complement pathway and (iii) zilucoplan that inhibits C5 cleavage into C5a and C5b, and prevents the C5b-dependent assembly of the MAC (approved for myasthenia gravis) might pave the way for novel therapeutic scenarios for PG management.

#### Interleukin-1 inhibitors

Consistent with the crucial role of IL-1 in neutrophils activation and inflammasome formation, IL-1 blockers including anakinra, an IL-1 receptor antagonist (IL-1RA) that blocks IL-1α and β, and canakinumab that inhibits the inflammatory activity of IL-1β, have been successfully used for treating PG, including its syndromic manifestations, improving cutaneous and extracutaneous PG lesions [18].

Canakinumab is the only drug in this class tested in a phase II open label trial [67], in which 4 out of 5 patients (80%) with steroid-resistant PG showed a decrease in primary ulcer size by week 16. Similarly, another study reported a complete remission of PG in 6 out of 11 patients and clinical improvement in 1 patient [68].

On the other hand, 10 of 12 patients (83.3%) treated with anakinra experienced either significant clinical improvement or complete resolution [68].

#### Interleukin-17 inhibitors

IL-17 inhibitors, including secukinumab (anti-IL-17 A) brodalumab (IL-17RA) and ixekizumab, an anti-IL-17 A/F, have been successfully reported as safe and effective choices for PG [69, 70].

A phase II open-label study demonstrated the efficacy of secukinumab in pain reduction in 4 out of 7 patients and significant clinical improvement with decrease of serum inflammatory markers in 2 patients within 32 weeks, although 3 out of these patients worsened [71].

An ixekizumab phase II study failed to meet its primary endpoint after 12 weeks of treatment (NCT03137160) [72], although a recent case series documented a complete resolution of PG in all 4 patients after 3 months of treatment.

Although trials on brodalumab are not available, recent evidence reported the complete clinical remission of PG in 3 patients by 12 weeks, one of whom was refractory to secukinumab [62].

Noteworthy, IL-17 antagonists have also been implicated in inducing PG, most likely due to a paradoxic increase in IL-23 [15, 73].

## Level 2 evidence (Cohort Studies)

### Dapsone

Dapsone is the most common immunomodulatory agent used in PG with anti-inflammatory, anti-bacterial and antibiotic properties. Although its exact mechanism of action is not well understood, it is considered an antineutrophilic agent that exerts its function by inhibiting myeloperoxidase in neutrophils and preventing tissue damage from reactive oxygen species (ROS) [32].

It is mainly used in combination with other systemic, topical and intralesional CS, antibiotics or TNF-α inhibitors; a retrospective study of 27 patients revealed a 96.9% response rate, of whom 81.3% had partial response and 15.6% achieved complete response after a mean time of 14.3 months. In spite of that, a third of patients developed AEs such as haemolytic anemia and methemoglobinemia in 9.4% and 3.7%, respectively [74].

### Mycophenolate mofetil

Mycophenolate mofetil (MMF) is an immunosuppressive agent and inhibitor of inosine-5’-monophosphate dehydrogenase, a rate-limiting enzyme in de novo synthesis of guanosine nucleotides. Since T- and B-lymphocytes are more dependent on this pathway, MPA acts preferentially in activated T and B lymphocytes by inhibiting their proliferation and suppressing cell-mediated immune responses and antibody formation.

It has been evaluated as a first-line or second-line steroid-sparing agent and used concomitantly with systemic CS in patients with PG [75]. In this retrospective study of 26 patients treated with MMF and prednisolone, 84.6% showed clinical improvement, of whom 50% achieved complete healing within an average time of 12.1 months. Unfortunately, 5.8% of patients experienced side effects, although most of them were mild (26.9%) [76].

The safety and efficacy of MMF has also been documented in the clinical setting of refractory PG or in patients who declined biologic treatments [69].

Overall, the use of MMF allowed patients to reduce their mean doses of systemic CS, appearing an efficacious and well-tolerated adjunctive therapy option for PG [62].

### Topical corticosteroids and intralesional corticosteroids

High-potency topical CS have been found to be effective mainly for the treatment of mild or localized PG. The most widely used is clobetasol propionate; in a prospective cohort study of 66 patients under treatment with clobetasol propionate 0.05% cream monotherapy, 43.8% of these patients healed by 6 months. Similarly, the use of intra-lesional steroid injections is widely recognized, often for the treatment of peristomal PG because it is administered intermittently when changing ostomy appliances and does not interfere with device adhesion [77].

Overall, caution is recommended with higher concentrations or injection volumes as skin atrophy is a common side effect.

#### Topical calcineurin inhibitors

Topical calcineurin inhibitors include tacrolimus, pimecrolimus, and cyclosporine that inhibit T-cells activation and proliferation and the secretion of various pro-inflammatory cytokines.

Topical tacrolimus ointment 0.1% resulted in complete ulcer healing in 50% to 100% of PG patients within 2 to 6 months, thus representing an effective first-line treatment for mild PG. It’s important to mention that renal injury was recorded after applying topical tacrolimus on open wounds [77].

In contrast, topical pimecrolimus, having less systemic absorption than topical tacrolimus, might be a safer choice for mild PG or as adjuvant therapy in moderate-to severe form of PG [62].

Similarly, topical cyclosporine, which has shown a remarkable response rate, could represent a promising option as it appears to be free of the tolerability problems associated with systemic administration [62].

Of note, topical calcineurin inhibitors are also recommended for an adequate control of wound bed and perilesional inflammation in the setting of wound care during the inflammatory phase [78].

#### Level 3 (Case-Control Studies) and Level 4 (Case Series or Case Reports) Evidence 

Table 4 [79–114] summarized the multiple treatments, in terms of conventional systemic and topical therapies, biologic agents and small molecules, supported by level 3 and 4 evidence, that have proven effective for the treatment of PG.

**Table 4:** Multiple treatments, supported by level 3 and 4 evidence, that have proven effective for the treatment of pyoderma gangrenosum

Drug	Mechanism of action	Current level of evidence	Clinical outcome
Conventional systemic and topical therapies			
Intravenous immunoglobulin	i) Neutralizes pathogenic circulating antibodies; ii) inhibits antibody production, complement activation and migration of inflammatory cells; iii) induces autoreactive T cells apoptosis	3	1) In addition to immunosuppressive therapy, 44 out of 50 patients (88%) had response rate; 53% of these PG patients achieved complete healing within 6 months without SAEs [79] 2) Of 45 PG patients refractory to different treatments, 51% of those healed completely [80]
Methotrexate	i) Inhibits cells division; ii) increases lincrNa-p21 expression, which regulates immune responses	4	1) Successfully used as a steroid-sparing agent in association with different conventional systemic therapies or anti-TNFα [18,81] 2) Intralesional MTX was also used as an adjuvant therapy to oral CS allowing patient to achieve almost complete ulcer healing by 7 weeks of therapy [82]
Azathioprine	Affects lymphocyte activation and antibody production	4	Successfully used as a steroid-sparing agent in combination with other conventional systemic and topical therapies as well as infliximab [83]
Colchicine	Prevents the activation, degranulation, and migration of neutrophils	4	Used as maintenance monotherapy [84]
Thalidomide	Modulates NF-κB-related proinflammatory cytokines and chemokine	4	1) It has been proposed as monotherapy or in combination with CS or dapsone [85] 2) Successfully used in paediatric PG population [86].
Topical timolol	Acts as a nonselective β-adrenoblocker	4	It has proven effective as an adjunct for localized/persistent PG, especially in non-inflammatory phase [87]
Topical phenytoin	Promotes fibroblast proliferation, collagen deposition, and neovascularization, while also reducing inflammation, edema, and bacterial load	4	6 patients showed a clinical improvement of PG lesions, although 5 were concomitantly treating with systemic therapy [88]
Granulocyte and monocyte adsorption apheresis	Extracorporeal treatment capable of removing activated myeloid lineage leukocytes, with the great potential to decrease inflammation and tissue damage	4	Successfully used, either as in monotherapy or in combination with systemic CS and biologics, in several cases of PG, including two PASH patients [62, 69, 89]
***Biologic agents***			
Ustekinumab	Selectively targets p40 subunit of IL-12 and IL-23	3	1) Of 34 PG patients, 79% experienced clinical improvement whereas 71% healed completely [90] 2) A case-control study of 28 patients reported a response rate of 68% for all clinical variants of PG [91]
Tocilizumab	Inhibits IL-6, which i) exerts stimulatory effects on T-and B cells; ii) activates intracellular signaling cascades through JAK1/STAT3 pathway; iii) favours a Th17-skewed inflammation; iv) participates in neutrophil activation and accumulation in inflammation site	4	1) Successfully used in a single patient with PG and concomitant RA [92] 2) Clinical improvement of PG in a patient diagnosed with Takayasu arteritis [93], although a paradoxical PG has also been reported after tocilizumab treatment for Takayasu arteritis [94]
i) Tildrakizumab; ii) Guselkumab iii) Risankizumab	Target the IL-23 p19 subunit	4	Significant clinical improvement or complete healing after 3 to 8 months of therapy; particular efficacy of guselkumab [95] and risankizumab [96] in patients with recalcitrant PG
Spesolimab	Blocks the receptor of IL-36, preventing the activation of downstream signaling pathways that promote inflammation	4	Successfully used (off-label prescription) in 3 patients with refractory PG: 1) One patient with post-operative PG experienced significant clinical improvement within 5 weeks [97] 2) In the second patient, a marked improvement of pain and inflammation as well as reduction in ulcer exudation within 48 h of spesolimab infusion occurred; complete healing of PG lesions after several weeks [97] 3) The third patient showed a complete resolution of all PG ulcers within 3–4 weeks of treatment [98]
Vedolizumab	Targets the α4β7 integrin, which specifies the recruitment of T cells	4	Five patients with IBD experienced an improvement of PG (in combination with both conventional systemic therapies [99,100] and biologics/small molecules [101,102] Of note, vedolizumab-induced PG has also been reported in IBD patients [103]
Visilizumab	Binds to the CD3 receptor on certain activated CD8 + and CD4 + naive T cells	4	A patient with PG and UC achieved complete healing after 6 months of therapy (5 mcg/kg/dose intravenously) [104]
Rituximab	Targets CD20 (expressed at most stages of B-cell development	4	It has proven safe and effective for the treatment of both PG-like ulcers in association with granulomatosis with polyangiitis [105] and refractory PG ulcers in the absence of associated conditions [106] Of note, it has been reported that it may be implicated in most cases of new-onset PG during biologic therapies [14]
Bortezomib	Inhibit the proteasome	4	Prompt healing in PG cases with concurrent hematologic diseases, such as IgA gammopathy of undetermined significance [107,108] and smoldering multiple myeloma [109]
Ixazomib	Inhibit the proteasome	4	Complete remission in just a few weeks since initiation of therapy in a case of PG with concurrent IgA smoldering multiple myeloma [110]
***Small molecules***			
Tofacitinib	Inhibits JAK1 and JAK3	4	Of 17 PG patients, 10 achieved complete resolution within a median of 12 weeks [111]
i) Ruxolitinib; ii) Baricitinib	Inhibit JAK1 and JAK2	4	1) Ruxolitinib (*n* = 2) appeared safe and effective in the treatment of PG associated with polycythemia vera [111] 2) Baricitinib (*n* = 5) has shown PG ulcer healing when used to treat underlying comorbidities [110]
Upadacitinib	Selectively targets JAK1	4	Complete remission (*n* = 4) with a median of 12 weeks [112]
Apremilast	Inhibits PDE-4, responsible for hydrolysing cAMP, which promotes the downstream expression of numerous pr-inflammatory cytokines and downregulates anti-inflammatory cytokines such as IL-10.	4	1) In one patient with 3-year history of recalcitrant vegetative PG, apremilast was added to systemic CS and MTX and complete healing was achieved at 4 months [113]. 2) One patient with 5-year history PG in the absence of comorbidities experienced clinical improvement by 4 months and complete remission after 3 years of therapy [114] 3) One PG patient with concomitant colonic CD showed a marked reduction of PG skin lesions after 19 weeks of therapy [101]

*SAE *serious adverse events,* lincrNa* p53-induced long noncoding RNA, *MTX* methotrexate, *CS* systemic corticosteroids, *NF-κB* nuclear factor kappa-light-chain-enhancer of activated B cells,* IL* interleukin, *RA* rheumatoid arthritis, *IBD* inflammatory bowel disease, *UC *ulcerative colitis, *JAK* Janus kinases, *STAT* signal transducer and activator of transcription proteins, *PD3E-4 *phosphodiesterase-4,* cAMP *cyclic adenosine monophosphate

Different biologics, small molecules and advanced therapies are also currently under investigation for the treatment of PG (Table 5) [29, 66, 120).

**Table 5:** Biologics, small molecules and advanced therapies currently under investigation for the treatment of pyoderma gangrenosum registered at ClinicalTrials.gov database

Therapeutic Agent	NCT identifier	Mechanism of action	Current phase of development	Notes (e.g., signals of effectiveness/safety)	Approved/commercially available (e.g., for other indications)	Reference
Vilobelimab	NCT03971643	mAb that blocks complement factor C5a	3	Phase 2a study (NCT03971643) demonstrated treatment response rates correlated with suppression of C5a levels in patients’ plasma over time.	COVID-19	66
Guselkumab	NCT06563323	mAb that blocks IL-23	2	Effectiveness has been reported in some case reports.	Plaque psoriasis, psoriatic arthritis, (ulcerative colitis)	115
Baricitinib	NCT04901325	Inhibitor of JAK1 and JAK2	2	Effectiveness has been reported in some case reports.	Rheumatoid arthritis, atopic dermatitis, alopecia areata, juvenile idiopathic arthritis	116
Deucravacitinib	NCT05821374	Inhibitor of TYK2	1	None	Plaque psoriasis	117
Autologous Platelet-Rich Plasma	NCT05984654	Activate the wound-healing cascade stimulating formation of new blood vessels and collagen in PG ulcers	NA	Effectiveness has been reported in some case reports.	Investigational use in chronic wounds and musculoskeletal conditions.	120
Dehydrated human amnion/chorion membrane	NCT05120726	Releases growth factors and anti-inflammatory interleukins, modulates MMP activity, promotes mesenchymal stem cell migration.	4	Effectiveness has been reported in some case reports.	Used for managing chronic wounds such as diabetic and venous ulcers.	121

*mAb* (Monoclonal Antibody), *IL* (Interleukin), *JAK* (Janus Kinase), *TYK2* (Tyrosine Kinase 2), *PG* (Pyoderma Gangrenosum), *MMP* (Matrix Metalloproteinases), *NA* (Not Applicable)

**Table 6:** Monthly estimated costs and levels of evidence for off-label biologic therapies for pyoderma gangrenosum

Biologic	Dosage per administration*	Dosing frequency*	Level of Evidence	Monthly cost (UK)**	Monthly cost (USA)**
Infliximab	5 mg/kg	Every 4–8 weeks	Level 1	£840-£1,680°	$2,480-$4,960°
Adalimumab	40 mg	Weekly	Level 1	£1,408	$14,596
Etanercept	25–50 mg	Weekly	Level 4	£716	$8,000
Anakinra	100 mg	Daily	Level 4	£810	$9,330
Canakinumab	150 mg	Single dose or more	Level 1	£9,928^	$20,484^
Secukinumab	150–300 mg	Every 1–4 weeks	Level 4	£609-£2,436	$7811-$31,244
Ixekizumab	80 mg	Every 4 weeks	Level 4	£1,125	$7,292
Brodalumab	210 mg	Weekly	Level 4	£2,760	$7,340
Ustekinumab	90 mg	Every 8–12 weeks	Level 3	£716-£1,074	$4,889-$7,335
Guselkumab	100 mg	Every 4–8 weeks	Level 4	£2,250-£4,500	$14,617-$29,345
Risankizumab	150 mg	Every 8–12 weeks	Level 4	£1,109-£2,218	$7,380 − 14,760
Tildrakizumab	100 mg	Every 8–12 weeks	Level 4	£1,080 − 2,160	$5,765 − 11,530
Vedolizumab	300 mg	Every 8 weeks	Level 4	£1,025	$4,567
Tocilizumab	162 mg	Every 2 weeks	Level 4	£456	$2,770
Spesolimab	900 mg	Single dose or more	Level 4	£15,000^	$54,929^

*Due to the off-label nature and lack of standardized dosing for PG, the dosage and the frequency for these biologics varies and is often tailored to individual patient needs

**Wholesale acquisition costs from manufacturers’ sites, drugs.com, and British National Formulary (last accessed on November 1 st, 2024)

°for a patient weighting 80 kg. Cost varies based on patient weight

^Cost per dose

#### Wound care

Wound care is essential to promote healing. PG-TIME (tissue, infection, moisture balance, and epithelialization) approach has been recently proposed as novel therapeutic algorithm in both the inflammatory and noninflammatory healing phases [78]. According to this approach, during the inflammatory phase, perilesional skin should be treated with medium-high grade steroids, pimecrolimus, and tacrolimus whereas the wound bed should be treated similarly in both inflammatory and noninflammatory healing phase with appropriate debridement (enzymatic with collagenases or autolytic with hydrogel) and adsorbent dressings such as alginate and hydrofiber. To address critical colonization, antiseptics are recommended. The last phase of epithelialization can be improved with bioactive dressings, allografts, epidermal grafts and skin substitutes [78]. Although further randomized controlled trials are needed to determine the most effective wound care regimen for the various clinical presentations of PG, several emerging strategies are proving effective, mainly including hyperbaric oxygen therapy, platelet-rich plasma and dehydrated human amnion/chorion membrane allografts.

The role of hyperbaric oxygen (HBO) therapy for wound healing is well known due to its effects on oedema reduction, inflammation control, collagen formation, and bacterial burden mitigation. However, there is very limited evidence documenting the success of treating PG with HBO therapy [118], and further larger studies are needed.

Platelet-rich plasma (PRP) is a volume of autologous plasma with a platelet concentration above baseline together with endogenous growth factors and cytokines able to promote new vessels and collagen formation. Its efficacy was demonstrated in diabetic foot ulcers and venous leg ulcers for which the probability of complete closure increased 5-fold compared with those treated with conventional treatments. Until now, 2 patients with PG have been successfully treated with 3–4 applications of PRP without any other topical or systemic therapy [119]. A prospective randomized, split ulcer-controlled trial is currently in progress to assess the efficacy and safety of PRP and whether PRP is more effective as topical solution than as injections [120].

Finally, dehydrated human amnion/chorion membrane (dHACM) allografts, - skin substitutes containing components of ECM that can be placed in wound beds to accelerate wound healing and decrease pain -, have shown promising results in PG treatment as well. A clinical trial is currently ongoing to evaluate the use of dHACM followed by skin grafting [121].

#### Perspectives

Current challenges to be faced in PG care include the need for accurate diagnostic techniques, especially for chronic cases, and effective therapeutics.

While the results of two pivotal trials are expected to provide robust evidence to guide PG management in the future, clinicians should not defer treatment initiation with off-label biologics or their combinations.

Among available treatment options, systemic corticosteroids and TNF-α inhibitors have the greatest body of evidence. In the meanwhile, anti-IL-12/23 and anti-IL-23 agents seem to offer an advantage in terms of safety, tolerability, and efficacy. In the Authors’ opinion, in the future they could be envisioned as backbone therapy, with other agents as addons to address the acute phase.

It is equally important to recognize that adequate treatment of background/associated diseases is instrumental to optimal PG management, both for informing drug choice based on comorbidities and to adequately target its pathogenesis. While this concept has been increasingly incorporated for IBD-associated cases as well as those linked to rheumatological conditions, a paradigm shift for PG related to haematological conditions is still needed. Currently, this is held back by the fear of commencing active treatment for premalignant haematological conditions. However, although certainly there are risks to be carefully discussed with the patients, eradicating the associated haematological neoplasm is sometimes the only way to cure PG and may be best done earlier in the course of the disease, before a decay in the patient status is observed.

In addition to the well-known diagnostic challenges in PG, the lack of standardized disease and patient outcome measures has complicated the monitoring of treatment response, making it difficult to compare the available treatments. The next steps of UPGRADE’s core outcome set (COS) development will involve domain items from existing literature, refining them through e-Delphi surveys until a final consensus is reached [28]. This project will define more accurate outcome measures in PG clinical trials, thus improving therapeutic strategies and patient care.

## Data Availability

All data generated or analysed during this study are included in this published article.
